# Description of a PCR-based technique for DNA splicing and mutagenesis by producing 5' overhangs with run through stop DNA synthesis utilizing Ara-C

**DOI:** 10.1186/1472-6750-5-23

**Published:** 2005-09-01

**Authors:** Menachem Ailenberg, Neil M Goldenberg, Mel Silverman

**Affiliations:** 1 Department of Medicine, Medical Science Building, Room 7207, University of Toronto Toronto, Ontario M5S 1A8, Canada

## Abstract

**Background:**

Splicing of DNA molecules is an important task in molecular biology that facilitates cloning, mutagenesis and creation of chimeric genes. Mutagenesis and DNA splicing techniques exist, some requiring restriction enzymes, and others utilize staggered reannealing approaches.

**Results:**

A method for DNA splicing and mutagenesis without restriction enzymes is described. The method is based on mild template-dependent polymerization arrest with two molecules of cytosine arabinose (Ara-C) incorporated into PCR primers. Two rounds of PCR are employed: the first PCR produces 5' overhangs that are utilized for DNA splicing. The second PCR is based on polymerization running through the Ara-C molecules to produce the desired final product. To illustrate application of the run through stop mutagenesis and DNA splicing technique, we have carried out splicing of two segments of the human cofilin 1 gene and introduced a mutational deletion into the product.

**Conclusion:**

We have demonstrated the utility of a new PCR-based method for carrying out DNA splicing and mutagenesis by incorporating Ara-C into the PCR primers.

## Background

Splicing of DNA molecules is an important task in molecular biology that facilitates cloning, mutagenesis and the creation of chimeric genes. While the advent of restriction enzymes substantially advanced DNA splicing techniques, they cannot be applied universally, and their use is limited to enzyme-specific loci. Other techniques like site-directed mutagenesis by overlap extension [SOE; [1]], insertional mutagenesis with the megaprimer technique [2] and staggered reannealing [3,4] have further improved DNA mutagenesis and splicing. Each method offers advantages and inherent drawbacks. Another cloning approach involving the formation of 5' overhangs utilizes incorporation of nucleotide derivatives into PCR primers [5-7] that stall polymerization. These techniques are dependent on an established set of optimal conditions for strong polymerization arrest, including the correct choice of polymerase or the incorporation of three ribonucleotide derivatives in the primer [7]. Furthermore, chimeric DNA/RNA primers need to be removed and reverse-transcribed in order for the splicing to be completed.

Although in the past we have successfully used the SOE technique for mutagenesis and splicing, we encountered difficulties while constructing larger genes. That led us to develop the staggered reannealing method [3,4]. This method proved to be useful as well, however, its efficiency declined as the gene to be mutagenized exceeded 1000 bp. Although these techniques allow splicing of any two DNA fragments without the need for restriction enzymes, their efficiency is inversely related to the length of the DNA fragments involved, since these techniques rely on the successful melting and reannealing of DNA to create matching overhangs. We sought to offer an alternative approach to facilitate the splicing of any two DNA segments for mutagenesis and construction of chimeric genes. Our technique utilizes two rounds of PCR, and is based on moderate template-dependent polymerization arrest using cytosine arabinose (Ara-C).

**Figure 1:**
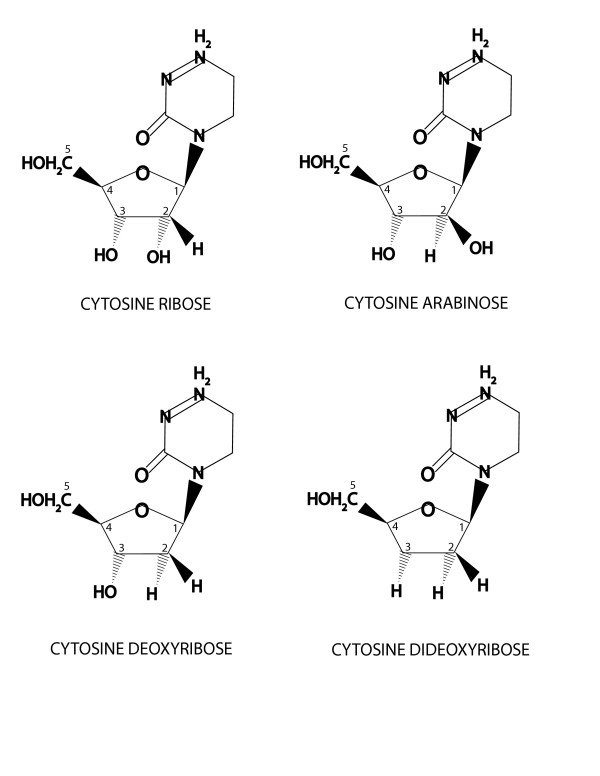
**Structure of cytidine and its derivatives**. The derivatives featured in this figure vary in their sugar substitutes. Note that in Cytosine Arabinose (Ara-C), the arabinose sugar contains hydroxyl groups in positions 3 and 5 in a similar orientation to native ribose, thus permitting reaction with other nucleotides in DNA synthesis. However, the hydroxyls in positions 2 and 3 are in the *trans *orientation. Comparing position 2 on the arabinose ring to that of 2-deoxyribose reveals that the hydrogen in 2-deoxyribose, that is in trans configuration to hydroxyl 3, is replaced by the hydroxyl group found on arabinose. It should be emphasized that there are two types of polymerization arrest: a. Chain termination- the nucleotide is incorporated into the nascent DNA strand and synthesis is stalled because no new nucleotide is added. Dideoxy derivatives stall elongation after incorporation into the nascent DNA strand because they do not have hydroxl in position 3. Arabinose nucleotides also belong to this group, but they offer only partial stalling [11]. b. Template-dependent termination-nucleotides already incorporated in the DNA (e.g. in primers) are able to stall polymerization when the polymerase reads the template. It is believed that due to stereo restraints, the polymerase falls off the template. The frequency of this event determines the efficiency of the stalling. Arabinose derivatives belong to this group. The property of template-dependent termination of Ara-C was utilized in this study to create 5' overhangs in the first PCR. However, since the template-dependent termination by Ara-C is moderate, it was utilized for the amplification in the second PCR.

Ara-C is a nucleotide derivative (Fig 1) that is widely used in cancer therapeutics [8]. It is a competitive inhibitor of DNA polymerase and also affects polymerization initiation [9,10]. Ara-C exerts it therapeutic action on cellular DNA polymerase after phosphorylation by an endogenous kinase. Once phosphorylated, Ara-C facilitates inhibition of DNA replication in cancer cells. Sanger et al, in their search for polymerization terminating agents for use in sequencing techniques, found that while dideoxynucleotides were strong polymerase terminators, Ara-C only weakly halted polymerization [11]. Therefore, even today, dideoxynucleotides remain the terminators of choice in sequencing reactions. Previous studies have shown that while Ara-C could serve as a substrate for mammalian polymerases, it terminates polymerization by some prokaryotic polymerases [11].

Here we used Ara-C both as DNA polymerase inhibitor and template for DNA mutagenesis and splicing.

## Results and discussion

**Figure 2:**
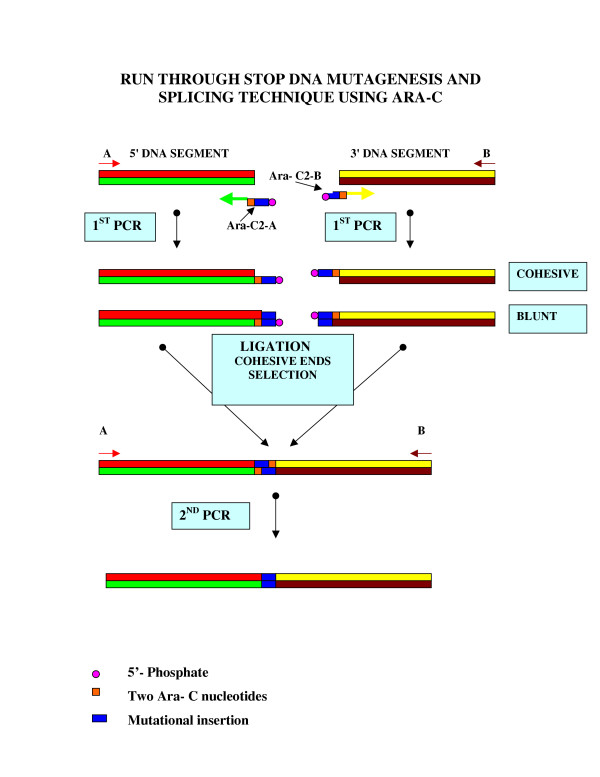
**Schematic representation of run through stop DNA mutagenesis and splicing technique with Ara-C**. In this example two pieces of DNA are to be spliced (5' and 3' DNA segments) and mutated with an insertion of additional DNA. The 5' segment is amplified using PCR primers A (sense) and Ara-C2-A (anti-sense). Primer Ara-C2-A is designed to contain hybrid DNA with two adjacent molecules of Ara-C to stall polymerisation and produce a 5' overhang. Mutational addition is also incorporated into this primer. (Note that in this paper we created a mutational deletion in the human cofilin 1 gene, but here for illustration purpose, we describe a mutational addition). The 3' segment is amplified using PCR primers Ara-C2-B (sense) and B (anti-sense). Primer Ara-C2-B contains overlapping sequence with primer Ara-C2-A, and 2 molecules of Ara-C are incorporated to stall polymerization and produce a 5' overhang that is complementary to the overhang in Ara-C2-A. Both Ara-C primers are phosphorylated for down stream ligation. Since two adjacent Ara-C molecules produce moderate termination, PCR products contain a mixture of 5' overhang and blunt end DNA. Each PCR product is gel-isolated and subjected to short ligation, where cohesive end ligation is predominant. A portion of the ligation reaction is then subjected to a second PCR reaction, using primers A and B that span the entire mutated chimeric DNA. As mentioned above, 2 Ara-C molecules are moderate polymerization terminators. This assures that at the first round of the second PCR, the polymerase will run through the Ara-C in the template and incorporate native dGMP, that will ensure in turn proper polymerization in the next rounds and a product that will contain the native dCMP. For cloning purposes of the final PCR product, primers A abd B can include restriction sites (as used in this study). Alternatively, by using Taq polymerase in the second PCR reaction, the product can be cloned into TA cloning plasmids. Another alternative is to design primers A and B to contain at least 2 molecules of Ara-C to produce 5' overhangs to match cloning plasmids.

We were searching for a mild template-dependent polymerization terminator. The rationale for *mild *termination is as follows: Termination must be strong enough to create 5' overhangs in the first PCR reaction, but weak enough to allow the polymerase to continue through the modified nucleotide during the second round of PCR (Fig 2). For the reasons mentioned above, Ara-C was chosen for use in the present study.

**Figure 3:**
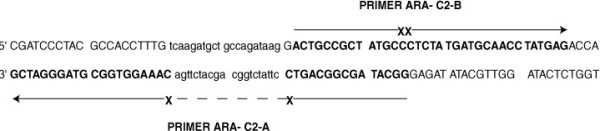
**Ara-C primer assignment**. Shown is the double-stranded DNA segment of human cofilin 1 gene that was used for mutagenesis. Capital letters and arrows represent primers containing Ara-C molecules. Lower case letters represent deleted nucleotides, achieved with primer Ara-C2-A (broken line). Xs in primers denote Ara-C molecules that replace the original deoxy cytidine molecules. Note that the 5' end of primer Ara-C2-A was designed to produce an overhang, that restricts ligation to the 3' segment of the PCR product (see also Fig 2) only if termination occurred after the first Ara-C molecule. The 5' segment of primer Ara-C2-B was designed to produce an overhang that restricts ligation to the 5' segment of the PCR product (see Fig 2) only if termination occurred after two Ara-C molecules. Additionally, two primers containing only one Ara-C molecule insertion were synthesized (not shown).

As proof of principal, a 20 bp deletion in the human cofilin 1 gene was created. We tried to splice together two segments of the gene: one 5' (237 bp) and one 3' (309 bp) segment (Fig 2). Primers were designed with one or two Ara-C molecules replacing native deoxycytidine nucleotides. When two Ara-C molecules were incorporated into the primer (Hospital for Sick Children, Toronto, Canada), template-dependent termination can potentially occur before, after one, or after two Ara-C molecules. Therefore, to determine the termination location, we designed one side of the overhang to accommodate termination after two Ara-C molecules, and the other side of the overhang to accommodate termination after one Ara-C molecule (Fig 3).

There were a total of 8 PCR reactions that included two Ara-C primers for each of the two segments, and the two polymerases (Taq and Pfu) for each set of primers. PCR products were gel-isolated. At this stage, gel-isolation is essential in order to remove any of the original plasmid that might serve as a template in the second PCR reaction. Alternatively, the original plasmid may be eliminated by digestion with DpnI, although this option is less recommended, since traces of undigested plasmid could affect the outcome of the second PCR reaction. Corresponding segments to be spliced were combined (total of four tubes) and ligated. As indicated above, the rationale for this technique is that Ara-C is a mild polymerization terminator, and therefore it will produce a mixture of cohesive and blunt ends. Hence, the reaction is expected to both terminate (producing sticky ends essential for the splicing phase) and run through the Ara-C (producing blunt ends; this feature is essential to the second PCR reaction). Therefore, lowered concentration of ligase and reduced ligation time were used to optimize conditions to favor cohesive end ligation. The products of the ligase reaction were amplified by the second PCR with Taq or Pfu polymerases using the sense primer A, and the anti-sense primer B, which span the cofilin 1 gene. This PCR reaction produced the expected 552 bp product (blunt end ligation, is expected to produce an extra duplicated piece of DNA of 15 bp). The PCR products were either sequenced directly, or cloned into a plasmid and then sequenced. Based on sequencing results, we observed that incorporation of two Ara-C nucleotides into the PCR primers yielded the expected product. This suggests that the two molecules of Ara-C provided the desired mild termination to produce a product with 5' overhangs, but also allowed the polymerase to run through during the 2^nd ^PCR. Furthermore, based on the design of the primers, the polymerization stalled both after the first and the second Ara-C molecule. Both 5 and 30 min incubations with DNA Ligase were sufficient to preferentially ligate the cohesive ends. This further suggests that two adjacent molecules of Ara-C produce 5' overhangs. Even though both 5 and 30 min ligations were successful in producing the desired product, it is not recommended to allow the reaction to proceed for a prolonged time, nor is it recommended to use high levels of ligase, since these conditions may facilitate blunt end ligation that may produce a mixture of the blunt and cohesive end products. Both Pfu and Taq polymerases were equally capable of producing termination products in the first PCR, while still running through the Ara-C in the second PCR. When one molecule of Ara-C was incorporated in the PCR primers, no termination could be observed, as seen by the addition of a 15 bp segment in the PCR product indicative of blunt end ligation. Even ligation for 5 min in reduced concentration of ligase failed to produce cohesive end ligation when only one Ara-C was employed.

The run through stop method utilizes a novel approach for DNA splicing and mutagenesis. While other mutagenesis techniques like SOE, megaprimer and staggered reannealing create matching overhangs after melting and reannealing, the run through stop method creates matching overhangs by polymerization arrest with Ara-C. We were motivated to design the Ara-C approach because we were not successful in creating gene mutations with the other techniques. Hence, the run through stop offers a good alternative to these techniques.

It has been previously demonstrated that utilizing abasic or RNA nucleotides like tetrahydrofuran derivative or 2-o-methyl ribonucleotide in PCR primers produced 5' overhangs that facilitated cloning of DNA fragments into plasmids [5-7]. These approaches were dependent on strong polymerization termination by the nucleotides. Our technique established the conditions for mild termination of DNA polymerization with two Ara-C molecules. This enables us to use the Ara-C-containing primers in two steps of PCR for DNA splicing and mutagenesis. Although in the present study we used relatively short segments of DNA for proof of principal (~500 bp of the human cofilin gene), this technique, unlike the staggered reannealing technique, is not limited to short DNA fragments. Since both rounds of PCR in the present study are based on conventional PCR, the length limit of the DNA fragments to be mutagenized is that of the PCR technique.

## Conclusion

The run through stop method can be summarized in four steps:

1. Amplify two segments of DNA to be spliced using PCR, with phosphorylated primers containing two adjacent molecules of Arabinose nucleotide with overlapping sequence.

2. Gel-isolate the two DNA products, combine and ligate.

3. Amplify the spliced product with flanking primers using PCR.

4. Clone the product into a plasmid.

## Methods

### First PCR

For the first PCR, 4 primers were designed: Primers A and B flanking the human cofilin 1 gene (Fig 2) and two primers containing Ara-C molecules (Figs 2 and 3)). Primer A-5'-ATActgcagATGGCC**GCT**GGTGTGGCTGTCTGTG-3'-sense primer of human cofilin 1. Lower case letters represent Pst I sequence and bold letters represent Ala to Ser mutagenesis for down stream usage. Primer Ara-C2-A-5'-GGCATAGCGGCAGTC**XX**AAAGGTGGCGTAGGGATCG-3'-anti-sense primer that contains two Ara-C molecules (**XX**) and designed to delete a 20 bp segment from the human cofilin 1 gene (Fig 3). Primer Ara-C2-B-5'-ACTGCCCGTTATGC**XX**TCTATGATGCAACCTATGAG-3'-sense primer that contains two Ara-C molecules (Fig 3). Additionally, two primers containing only one Ara-C molecule insertion were synthesized. Primer B-5'-CAActcgagGGCTGCCAGATGCTCCAGGCAGG-3'-anti-sense primer of the 3' end of human cofilin 1 gene. Lower case letters represent the sequence for the Xho I gene. In the first PCR, Primer A was used with primer Ara-C2-A, and Primer Ara-C2-B was used with primer B. In the second PCR, primer A was used with primer B (see also Fig 2).

Ara-C primers were phosphorylated for 30 min at room temperature using T4 polynucleotide kinase (Invitrogen, Burlington, ON), followed by inactivation at 65°C for 10 min, and used for PCR with no further purification. PCR was performed with corresponding primers (see above and Fig 2, 3) using 1 U Pfu polymerase (Stratagene, La Jolla, CA) or 1 U of Taq polymerase (Sigma, Oakville, ON), and plasmid pOTB7 containing the human cofilin 1 gene as template (ATCC, Manassas, VA). PCR conditions were as follows: heating to 94°C for 5 min; 40 cycles of: 94°C, 55°C and 72°C each for 30 seconds; final elongation for 7 min. PCR products were gel-isolated using MinElute Plasmid Purification Kits (Qiagen, Mississauga, ON). Corresponding segments to be spliced were combined (total of four tubes) and ligated for 30 min with 400 U, or five min with 200 U of T4 ligase (NEB, Pickering, ON) followed by inactivation for 10 min at 65°C.

### Second PCR

Two μl of the ligase reaction were amplified by the second PCR with Taq or Pfu polymerase using the sense primer A, and the anti-sense primer B. Conditions for the second PCR were similar to those of the first PCR. The PCR products were purified (Qiagen). Alternatively, the PCR products were subjected to double digestion with PstI and XhoI followed by ligation into plasmid pcDNA3.1Zeo+ (Invitrogen). One μl of ligation reaction was used to transform 20 μl competent cells (DH5α; Invitrogen), using a short procedure: competent cells were incubated for 5 min on ice, and heat-shocked by immediate plating on pre-warmed (37°C) agar plates. Plasmids were prepared using Fast Plasmid Mini Kit (Eppendorf, Mississauga, ON), and sequenced using the T7 primer.

### DNA sequencing

The products of PCR, as well as the products that were cloned into plasmid pcDNA3.1Zeo+ were sequenced in both directions, utilizing primers A and B, or primer T7, respectively (Hospital for Sick Children).

## Authors' contributions

MA conceived and designed the study, performed the experiments and drafted the manuscript. NMG carried out some of the experiments, participated in critical evaluation and drafted the manuscript. MS provided general guidance, coordination and funding for the study, and drafted the manuscript.
